# Quantum chemical studies on hydrogen bonds in helical secondary structures

**DOI:** 10.1007/s12551-022-01034-5

**Published:** 2023-01-06

**Authors:** Yu Takano, Hiroko X. Kondo, Haruki Nakamura

**Affiliations:** 1 Graduate School of Information Sciences, Hiroshima City University, Hiroshima, 731-3194 Japan; 2 Institute for Protein Research, Osaka University, Suita, 565-0871 Japan; 3 Faculty of Engineering, Kitami Institute of Technology, Kitami, 090-8507 Japan; 4 RIKEN Center for Biosystems Dynamics Research, Suita, 565-0874 Japan

**Keywords:** Hydrogen bond, Secondary structure, Density functional theory, Negative fragment approach, α-Helix, 3_10_-Helix

## Abstract

We present a brief review of our recent computational studies of hydrogen bonds (H-bonds) in helical secondary structures of proteins, α-helix and 3_10_-helix, using a Negative Fragmentation Approach with density functional theory. We found that the depolarized electronic structures of the carbonyl oxygen of the *i*th residue and the amide hydrogen of the (*i* + 4)th residue cause weaker H-bond in an α-helix than in an isolated H-bond. Our calculations showed that the H-bond energies in the 3_10_-helix were also weaker than those of the isolated H-bonds. In the 3_10_-helices, the adjacent N–H group at the (*i* + 1)th residue was closer to the C=O group of the H-bond pair than the adjacent C=O group in the 3_10_-helices, whereas the adjacent C=O group at the (*i* + 1)th residue was close to the H-bond acceptor in α-helices. Therefore, the destabilization of the H-bond is attributed to the depolarization caused by the adjacent residue of the helical backbone connecting the H-bond donor and acceptor. The differences in the change in electron density revealed that such depolarizations were caused by the local electronic interactions in their neighborhood inside the helical structure and redistributed the electron density. We also present the improvements in the force field of classical molecular simulation, based on our findings.

**Supplementary Information:**

The online version contains supplementary material available at 10.1007/s12551-022-01034-5.

## Introduction

Proteins are found in all living organisms and are involved in almost all biological activities such as catalysis, molecular recognition, and material transport (Liljas et al. 2017; Branden and Tooze 2012). Since protein functions are strongly correlated with their three-dimensional structures, understanding the three-dimensional structure of proteins and their dynamic behaviors is essential for various scientific fields including chemistry, biology, medicine, agriculture, and food industry.

Proteins are biopolymers consisting of a large number of amino acids held together by peptide bonds. Protein structures are hierarchical, with distinct levels of structures (Holde Van et al. 1998), which represent increasing levels of complexity and include primary, secondary, tertiary, and quaternary structures. Secondary structure is the local and regular structure of a protein, including α- and 3_10_-helices, β-strands, and β- and γ-turns. These secondary structures are properly assembled to form tertiary structures. Thus, secondary structures can be considered the building elements of protein structures.

In secondary structures, a helix (e.g., α-helix and 3_10_-helix) is a group of residues that repeatedly rotate and rise along an axis. It is the most observed secondary structure. We can classify it into different helical conformations. The α-helix is found in 31% of all secondary structures and is the most widely recognized helical structural element in fibrous and globular proteins (Barlow and Thornton 1988). The second most common helical structure is the 3_10_-helix, which occupies 4% of the secondary structures.

These helices are stabilized by hydrogen bonds (H-bonds) formed between the amide hydrogens (the H-bond donors) and the carbonyl oxygens (the H-bond acceptors) of peptide bonds. H-bonds are one of the most important noncovalent interactions for chemical and biological phenomena (Saleh et al. 2012; Tantardini 2019; Tantardini et al. 2020). The significance of H-bonds in the secondary structures of proteins was recognized early (Pauling et al. 1951; Eisenberg 2003). The α-helix has 3.6 residues per turn and is a right-handed helix. In the α-helix, a H-bond is formed between the peptide carbonyl group at residue *i* and the peptide amino group at residue *i* + 4. In contrast, the 3_10_-helix has three residues per turn and is a right-handed helix. It has a H-bond between the peptide carbonyl group at residue *i* and the peptide amino group at residue *i* + 3, resulting in a tighter packing of the backbone compared with the α-helix (Fig. S1). Many computational chemists have studied H-bonds in secondary structures at various levels of theoretical depth. Wieczorek and Dannenberg (2003a, b) investigated H-bond cooperativity and the energetics of α-helices, suggesting that various factors contribute to their stability. Morozov et al. (2006) evaluated the origin of cooperativity in forming α-helices. Wu and Zhao (2001) studied the role of cooperativity in the formation of α-helices by performing theoretical calculations on α-helix models constructed using a simple repeating unit method. Parthasarathi et al. (2007) studied H-bond interactions in an α-helix model using the atom-in-molecules method. Ismer et al. (2008) investigated the temperature dependence of the stability of α-, π-, and 3_10_-helices compared with a fully extended structure using density functional theory (DFT) and harmonic approximation. However, a simple physicochemical theory accounting for helical secondary structural features of proteins is still immature.

An accurate and quantitative evaluation of H-bonds is also important for molecular dynamics (MD) simulations to investigate the dynamical behavior and folding process of proteins. Historically, it has been noticed since many years ago that each force field used in classical MD simulations shows a specific tendency to form an α-helix or a β-strand. For example, the AMBER C96 force field, which was developed just after the original AMBER force field (Cornell et al. 1995), preferred extended structures contrast to the α-helical preference of the latter one as mentioned by Kollman et al. (1997). This phenomenon has been repeatedly reported by many authors (Sakae and Okamoto 2003; Yoda et al. 2004a, b; Best et al. 2008; Best and Hummer 2009; Piana et al. 2011). Usually, this preference for force fields on the secondary structure formation is not a significant problem in the MD simulations of rigid globular protein structures. However, it has become a critical issue in understanding functionally important conformational changes (Higo et al. 2011; Shirai et al. 2014; Chebaro et al. 2015; Nishigami et al. 2016) in the folding simulations of flexible disordered regions (Higo et al. 2011; Chebaro et al. 2015) and long loops between secondary structures (Shirai et al. 2014; Nishigami et al. 2016). Yoda et al. (2004a, b) performed MD simulations to compare the secondary structural properties of commonly used force fields, finding that MD simulations with the AMBER ff94 (Cornell et al. 1995) and ff99 (Wang et al. 2000) force fields were in remarkable agreement with experimental data for α-helical polypeptides but not for β-hairpin polypeptides. Numerous attempts have been made to overcome this problem, such as increasing the torsional energies, rearrangements (Kamiya et al. 2005; Buck et al. 2006; Fujitani et al. 2009; Robustelli et al. 2018), and developing polarized charge models (Patel and Brooks 2004; Lopes et al. 2009). Regardless, the reasons behind the use of these methods remain unclear, and elucidation requires understanding the energy of hydrogen bonding in the secondary structure.

Here, we briefly review our computational studies of H-bonds in helical secondary structures of proteins, α-helix and 3_10_-helix, using a Negative Fragmentation Approach (NFA) with DFT (Kondo et al. 2019, 2022). We will also discuss the modification of the force field, approximating the H-bond energies, revealed by our findings.

## Model constructions and computational method

### Model constructions

We constructed whole-helix (WH) models of an α-helix and a 3_10_-helix, denoted as WH_alpha_-*n* and WH_3_10_-*n* models, respectively. In the model construction, we used poly-alanine amino acids capped with an acetyl group (ACE) and an *N*-methyl amide group (NME), denoted as ACE–(Ala)_*n*_–NME (*n* = 2–7 for the 3_10_-helices and *n* = 3–8 for the α-helices). The backbone torsion angles, *φ* and *ψ*, of the WH_alpha_-*n* and WH_3_10_-*n* models were set to their ideal values, as described in biochemistry textbooks, namely, *φ* =  − 57° and *ψ* =  − 47° for the WH_alpha_-*n* models and *φ* =  − 49° and *ψ* =  − 26° for the WH_3_10_-*n* models (Arnott and Dover 1967; Petsko and Ringe 2004; Kuster et al. 2015). These structures were optimized in the gas phase by fixing the backbone dihedral angles at the aforementioned values and minimizing the total electronic energies. One to six backbone H-bonds exist in the optimized WH_alpha_-*n* and WH_3_10_-*n* models. The *s*th H-bond in these models, counting from the *N*-terminus, is represented by *n*-*s*.

**Fig. 1:**
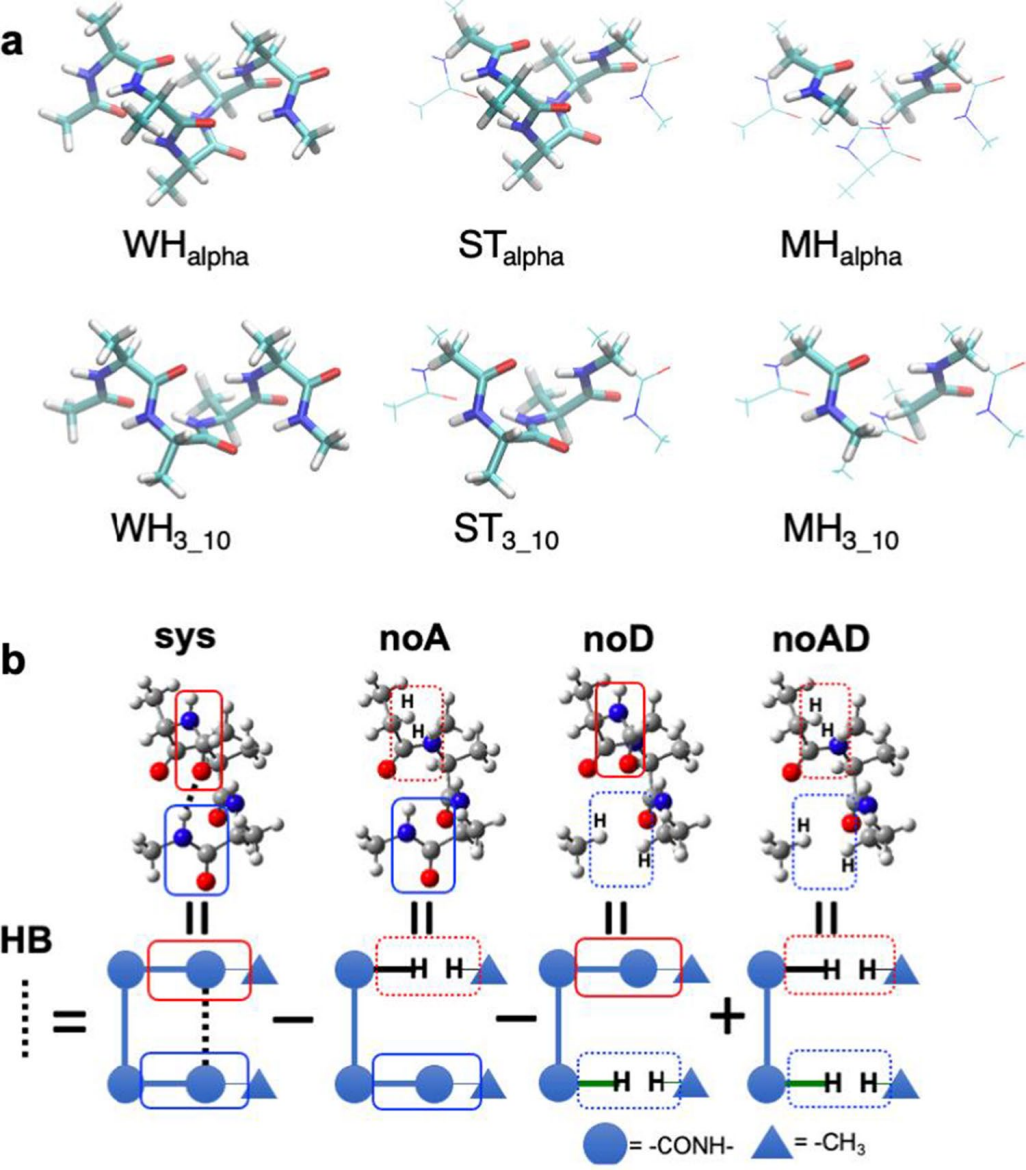
**a** Structures of the WH_alpha_-5, ST_alpha_-5, and MH_alpha_-5 models for an α-helix and those of the WH_3_10_-4, ST_3_10_-4, and MH_3_10_-4 models for a 3_10_-helix as examples. **b** Fragment structures and schematic picture for calculations of H-bond energy of 3–1 of the WH_alpha_-3 model with NFA

To understand the characteristics of the H-bond energies in α-helices and 3_10_-helices, we constructed two types of simplified models. One is a single-turn (ST) model, denoted as the ST_alpha_-*n* and ST_3_10_-*n* model composed of ACE-(Ala)_3_-NME and ACE-(Ala)_2_-NME, respectively. In this model, the H-bond donor and acceptor are linked with the helical backbone atoms. The other is a minimal H-bond (MH) model, denoted as MH_alpha_-*n* and MH_3_10_-*n* models comprising two separated *N*-methyl acetamide molecules and mimicking a single H-bond between the C=O and N–H groups in the backbone. In the MH_alpha_-*n* and MH_3_10_-*n* models, the two peptide groups forming a H-bond, hydrogen donors and acceptors, were separated without linking the helical backbone atoms. The atomic positions of these simplified models were the same as those of the corresponding WH_alpha_ and WH_3_10_ models, except for the N- and C-terminal capping groups. Figure 1a shows the molecular structures of the WH_alpha_-5, ST_alpha_-5, and MH_alpha_-5 models for an α-helix and those of the WH_3_10_-4, ST_3_10_-4, and MH_3_10_-4 models for a 3_10_-helix as examples. The individual H-bond energy for these models was calculated in the same manner as that for each backbone H-bond in the WH models, as described below. The H-bond energies of these models were then compared to each other.

### Validity of DFT exchange–correlation functionals for the calculation of H-bond energy of secondary structure

In recent years, DFT has become accepted as an alternative approach for the post Hartree–Fock (HF) methods such as Møller–Plesset perturbation theory (Head-Gordon et al. 1988) and coupled cluster theory (Scuseria and Schaefer 1989). In previous studies (Takano et al. 2011, 2012), we showed the importance of assessing the validity of various DFT exchange–correlation functionals. The DFT exchange–correlation functionals was also validated for the H-bond energies of the ACE-(Ala)_*n*_-NME system. We chose the B97D (B97 functional with Grimme’s D2 dispersion schemes) exchange–correlation functional (Grimme 2006) with 6–31+G(d) basis sets because it provided the H-bond energies of an ACE–(Ala)_*n*_-NME dimer comparable with the MP2 method in the calculation of the H-bond interaction energies of the ACE–(Ala)_*n*_-NME system in the gas phase (Takano et al. 2016). This method was applied to H-bond energy calculations of the helical secondary structures composed of ACE–(Ala)_*n*_-NME.

## Negative fragmentation approach

Since it is not straightforward to calculate a H-bond energy of a molecule, where the donor and acceptor atoms are linked through covalent bonds, we utilized the NFA, which is the modified version of the Molecular Tailoring Approach (MTA) developed by Deshmukh and Gadre (2009). As shown in Fig. 1b, each H-bond energy, ${E}_{\mathrm{HB}}$, in ACE-(Ala)_*n*_-NME is calculated by the following equation in the NFA:

1 $${E}_{\mathrm{HB}}={E}_{\mathrm{sys}}-{E}_{\mathrm{noA}}-{E}_{\mathrm{noD}}+{E}_{\mathrm{noAD}}$$

${E}_{\mathrm{sys}}$, ${E}_{\mathrm{noA}}$, ${E}_{\mathrm{noD}}$, and ${E}_{\mathrm{noAD}}$ represent the total electronic energy of the entire system, the system lacking the acceptor group, the system without the donor group, and the system lacking both the acceptor and donor groups, respectively.

In the NFA, we used the total electronic energy of the entire system $\left({E}_{\mathrm{Total}}\right)$ as ${E}_{\mathrm{sys}}$ (Kondo et al. 2019, 2022), while the energy of the entire system was estimated using the energies of all fragments in the original MTA (Deshmukh and Gadre 2009). The total energies of the WH_alpha_-*n* models estimated by MTA $\left({E}_{\mathrm{MTA}}\right)$ coincided well with the ${E}_{\mathrm{Total}}$ values of these models. The differences in the calculated values of ${E}_{\mathrm{Total}}$ and ${E}_{\mathrm{MTA}}$, ${E}_{\mathrm{MTA}}-{E}_{\mathrm{Total}}$, were less than 0.09 kcal/mol. These differences are similar to that obtained in the previous study (Deshmukh and Gadre 2009) for the 3_10_-helix (0.11 kcal/mol).

In addition to the H-bond energies, the NFA can approximately represent the change of electronic structures upon H-bond formation. The change in electron density upon H-bond formation, ${\Delta \rho }_{\mathrm{HB}}$, was evaluated as follows (Kondo et al. 2019, 2022):

2 $${\Delta \rho }_{\mathrm{HB}}={\rho }_{\mathrm{sys}}-{\rho }_{\mathrm{noA}}-{\rho }_{\mathrm{noD}}+{\rho }_{\mathrm{noAD}}$$

To examine the difference in the H-bond energy between the WH and MH models and between the ST and MH models in the context of their electronic structures, the differences in the change in electron density were computed using Eqs. 3 and 4, respectively:

3 $$\mathrm{\Delta \Delta }{\rho }_{\mathrm{HB}}^{\mathrm{WH}-\mathrm{MH}}=\Delta {\rho }_{\mathrm{HB}}^{\mathrm{WH}}-\Delta {\rho }_{\mathrm{HB}}^{\mathrm{MH}}$$

4 $$\mathrm{\Delta \Delta }{\rho }_{\mathrm{HB}}^{\mathrm{ST}-\mathrm{MH}}=\Delta {\rho }_{\mathrm{HB}}^{\mathrm{ST}}-\Delta {\rho }_{\mathrm{HB}}^{\mathrm{MH}}$$

As an advantage of NFA, no modifications of program code are required, though multiple calculations are needed. It indicates that we can utilize the NFA with any quantum chemical calculation programs.

For comparison and improvement of the force field, we also computed the H-bond energies based on the molecular mechanics (MM) with the AMBER ff99SB force field parameters (Wang et al. 2000), ${E}_{\mathrm{HB}}^{\mathrm{MM}}$, for the corresponding H-bonds as in the following equation:

5 $${E}_{\mathrm{HB}}^{\mathrm{MM}}=\sum \nolimits_{i,j \in \left\{\mathrm{C},\mathrm{ O},\mathrm{ N},\mathrm{\;and\;H}\right\}}\frac{{{q}_{i}q}_{j}}{{r}_{ij}}+\sum \nolimits_{i,j \in \left\{\mathrm{C},\mathrm{ O},\mathrm{ N},\mathrm{\;and\;H}\right\}}\left(\frac{{A}_{ij}}{{r}_{ij}^{12}}-\frac{{B}_{ij}}{{r}_{ij}^{6}}\right)$$

where *i* and *j* are the atoms constituting the peptide group of an acceptor and a donor of an H-bond, respectively: {C, O, N, and H}. *A*_*ij*_ and *B*_*ij*_ are the Lennard–Jones coefficients, *r*_*ij*_ is the distance between atoms *i* and *j*, and *q*_*i*_ is the atomic partial charge of the atom *i*.

## H-bond energies in helical model systems

**Fig. 2:**
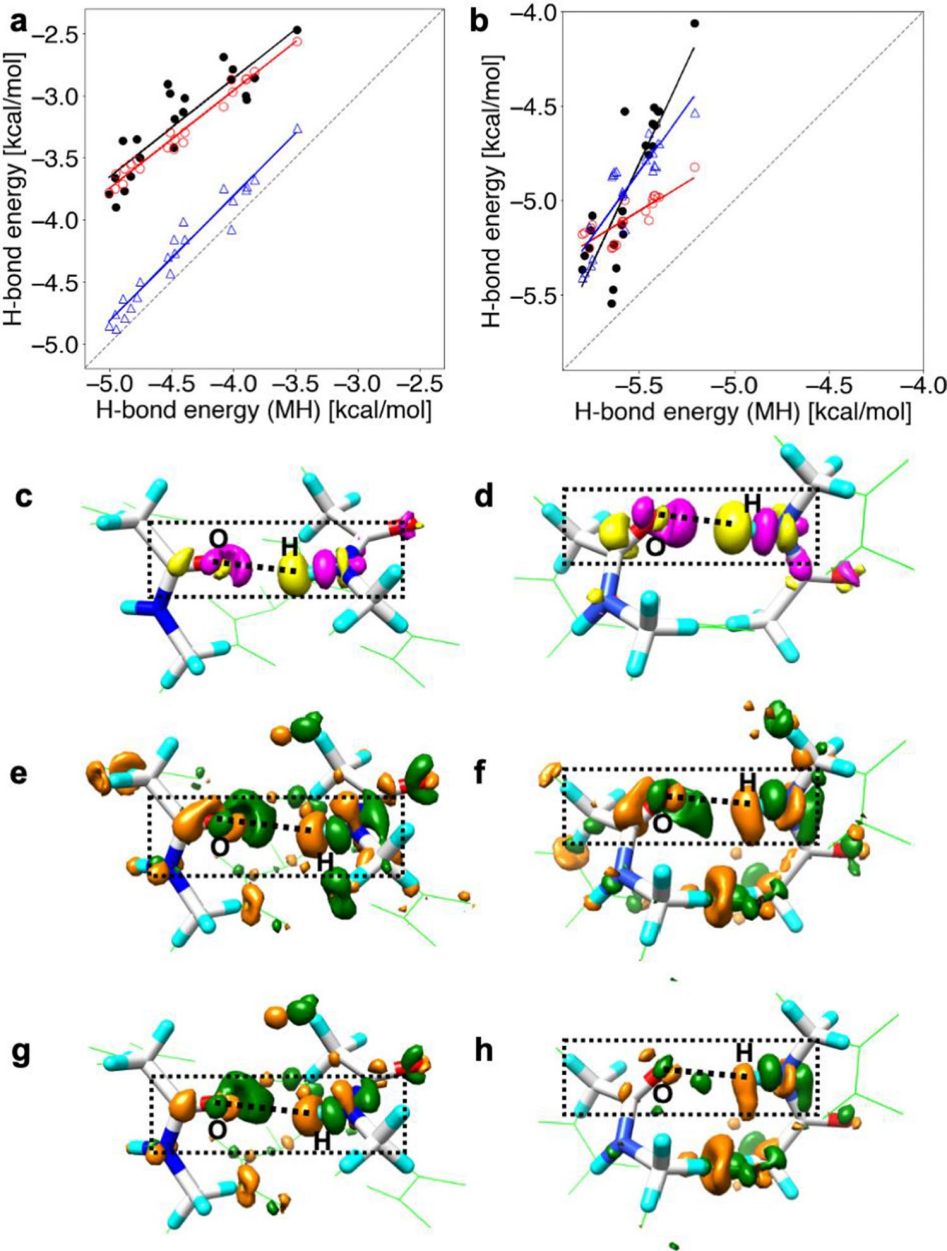
Correlations of the H-bond energies of the WH (black filled circle), ST (red open circle), and MM (blue open triangle) models versus those of the MH model in **a** the α-helices and **b** the 3_10_-helices. The dashed line shows a guide where the longitudinal axis values have identical H-bond energies (Kondo et al. 2022). Electron density changes upon H-bond formation, **c** $\Delta {\rho}_{\mathrm{HB}}^{{\mathrm{WH}}_{\mathrm{alpha}}}$ and **d** $\Delta {\rho}_{\mathrm{HB}}^{{\mathrm{WH}}_{3\_10}}$, for 5-2 of the WH_alpha_-5 model and for 4-2 of the WH_3_10_-4 model. The yellow surfaces represent the contour surfaces at −0.001 au, and the magenta ones are those at +0.001 au. The atoms in the whole WH models are shown by green wire and those in the MH models are shown using the stick model with CPK colors. The difference in the change in electron density between the WH and ST models, $\Delta \Delta {\rho}_{\mathrm{HB}}^{{\mathrm{WH}}_{\mathrm{alpha}}-{\mathrm{MH}}_{\mathrm{alpha}}}$ for **e** 5-2 of the WH_alpha_-5 model and $\Delta \Delta {\rho}_{\mathrm{HB}}^{{\mathrm{WH}}_{3\_10}-{\mathrm{MH}}_{3\_10}}$ for **f** 4-2 of the WH_3_10_-4 model. The difference in the change in electron density between the ST and MH models, $\Delta \Delta {\rho}_{\mathrm{HB}}^{{\mathrm{ST}}_{\mathrm{alpha}}-{\mathrm{MH}}_{\mathrm{alpha}}}$ for **g** 5-2 of the WH_alpha_-5 model and $\Delta \Delta {\rho}_{\mathrm{HB}}^{{\mathrm{ST}}_{3\_10}-{\mathrm{MH}}_{3\_10}}$ for **h** 4-2 of the WH_3_10_-4 model. The dark-green surfaces are the contour surfaces at −0.00015 au, and the orange ones are those at +0.00015 au. The black dotted line is the H-bond between the oxygen atom of the C=O group at the *i*th residue and the hydrogen atom of the N-H group at the (*i* + 4)th residue. The corresponding hydrogen bond is surrounded by a dashed rectangle. Figures **d**, **f**, and **h** were slightly modified from the original figures, which appeared in the previous paper by Kondo et al. (2022)

In order to understand the effects of the helical secondary structures on the H-bond energies, we compared the H-bond energies for the WH_alpha_-*n*, ST_alpha_-*n*, and MH_alpha_-*n* models of α-helices and the WH_3_10_-*n*, ST_3_10_-*n*, and MH_3_10_-*n* models of the 3_10_-helices. Since the H-bond energies strongly depend on the spatial arrangement of the H-bond donor and acceptor atoms, we plotted the H-bond energies of the WH and ST models against those of the MH models for the α- and 3_10_-helices in Fig. 2a and b, respectively, to cancel out the effect of the orientations of H-bond donor and acceptor (Kondo et al. 2022).

The H-bond energies obtained by the WH-*n* and ST-*n* models, ${E}_{\mathrm{HB}}^{\mathrm{WH}}$ and ${E}_{\mathrm{HB}}^{\mathrm{ST}}$, remarkably deviated from those calculated by the MH models. In the α-helices, the ST_alpha_-*n* model reproduced the H-bond energies of the WH_alpha_-*n* model. In contrast, the MH_alpha_-*n* model provided more stable H-bond energy than the WH_alpha_-*n* model (Fig. 2a), indicating that the adjacent residues covalently connecting the H-bond donor and acceptor destabilized the H-bond in the WH_alpha_-*n* model. In the 3_10_-helices, the ST_3_10_-*n* models also destabilized the H-bond as well as the α-helices compared to the MH_3_10_-*n* models but failed to provide the equivalent H-bond energies of the WH_3_10_-*n*. In particular, the H-bond pairs adjacent to the N- or C-terminal H-bond of the WH_3_10_-*n* models were strongly destabilized compared to those of the ST_3_10_-*n* models, resulting in the worse correlation of the WH_3_10_-*n* and ST_3_10_-*n* models. Details are discussed in our previous study (Kondo et al. 2022). It suggests that the destabilization of the H-bond of the 3_10_-helices is partly due to the helical backbone atoms linking the H-bond donor and acceptor. However, there should also be other factors leading to unstable H-bond energies.

We compared QM (${E}_{\mathrm{HB}}^{\mathrm{WH}}$) and MM (${E}_{\mathrm{HB}}^{\mathrm{MM}}$) calculations for the H-bond energies of the WH_alpha_-*n* and WH_3_10_-*n* models (Fig. 2a, b). In the WH_alpha_-*n* models, the ${E}_{\mathrm{HB}}^{\mathrm{WH}}$ values strongly correlated with the ${E}_{\mathrm{HB}}^{\mathrm{MM}}$ values, but the MM calculations overestimated the stability of the H-bond energies by ~ 1 kcal/mol. The ${E}_{\mathrm{HB}}^{\mathrm{MM}}$ values of the WH_alpha_-*n* models almost coincided with the H-bond energies calculated by the MH models, ${E}_{\mathrm{HB}}^{\mathrm{MH}}$. This is because the current force field parameters of amino acids are adjusted based on the amino acid monomer. In contrast, the H-bond energies evaluated with the QM calculations were closer to those with the MM calculations for the WH_3_10_-*n* models than for the WH_alpha_-*n* models. However, the correlation between the QM and MM calculations was weak, unlike the WH_alpha_-*n* models. In contrast to α-helices, the ${E}_{\mathrm{HB}}^{\mathrm{MH}}$ values were more stable than the ${E}_{\mathrm{HB}}^{\mathrm{MH}}$ values in 3_10_-helices. This is because the H-bonds in the MH_3_10_-*n* are much shorter than those in the MH_alpha_-*n* models, thus having stronger quantum nature that the classical force field cannot describe.

## Electronic structures around the H-bond donors and acceptors

The electron density changes for the WH_alpha_-5 and WH_3_10_-5 models, $\Delta {\rho }_{\mathrm{HB}}^{{\mathrm{WH}}_{\mathrm{alpha}}}$ and $\Delta {\rho }_{\mathrm{HB}}^{{\mathrm{WH}}_{3\_10}}$, were calculated with Eq. 2 and are shown in Fig. 2c and d, respectively. Here, yellow and magenta colors show the negative and positive contour surfaces, respectively. From the $\Delta {\rho }_{\mathrm{HB}}^{{\mathrm{WH}}_{\mathrm{alpha}}}$ values, the electron density increased around the oxygen atom of the C=O group at the *i*th residue and decreased around the hydrogen atom of the N–H group at the (*i* + 4)th residue, as shown in Fig. 2c. The $\Delta {\rho }_{\mathrm{HB}}^{{\mathrm{WH}}_{3\_10}}$ values showed that the electron density increased in the vicinity of the oxygen atom of the C=O group at the *i*th residue and that it decreased in the vicinity of the hydrogen atom of the N–H group at the (*i* + 3)th residue, as illustrated in Fig. 2d. The $\Delta {\rho }_{\mathrm{HB}}^{{\mathrm{WH}}_{\mathrm{alpha}}}$ and $\Delta {\rho }_{\mathrm{HB}}^{{\mathrm{WH}}_{3\_10}}$, thus, implied the formation of the H-bond.

In Fig. 2e and g, the differences in electron density change between the WH_alpha_-5 and MH_alpha_-5 models and between the ST_alpha_-5 and MH_alpha_-5 models, $\mathrm{\Delta \Delta }{\rho }_{\mathrm{HB}}^{{\mathrm{WH}}_{\mathrm{alpha}}-{\mathrm{MH}}_{\mathrm{alpha}}}$ and $\mathrm{\Delta \Delta }{\rho }_{\mathrm{HB}}^{{\mathrm{ST}}_{\mathrm{alpha}}-{\mathrm{MH}}_{\mathrm{alpha}}}$, respectively, were shown for the α-helical turn 5-2. Here, green and orange colors show the negative and positive contour surfaces, respectively. The electron density changes near the oxygen atom of the C=O group at the *i*th residue in both the WH_alpha_ and ST_alpha_ models were smaller than that in the MH_alpha_ model, implying that the negative polarization of the oxygen atom was weakened by the backbone atoms linking the H-bond donor and acceptor. In contrast, the electron density near the hydrogen atom of the N–H group at the (*i* + 4)th residue increased in the WH_alpha_ and ST_alpha_ models, as compared with that in the MH_alpha_ model. In addition, the $\mathrm{\Delta \Delta }{\rho }_{\mathrm{HB}}^{{\mathrm{WH}}_{\mathrm{alpha}}-{\mathrm{MH}}_{\mathrm{alpha}}}$ value was similar to the $\mathrm{\Delta \Delta }{\rho }_{\mathrm{HB}}^{{\mathrm{ST}}_{\mathrm{alpha}}-{\mathrm{MH}}_{\mathrm{alpha}}}$ values. It indicates that the weaker positive polarization of the hydrogen atom is mainly due to the adjacent residues connecting the H-bond donor and acceptor. We found that the distances between the oxygen atoms in the carbonyl group of the *i*th and (*i* + 1)th residues in the H-bond pairs were short (3.510 ± 0.144 Å). In addition, those between the hydrogen atoms in the amide group of the (*i* + 3)th and (*i* + 4)th residues were also short (2.676 ± 0.038 Å). These short distances caused the depolarization of both the carbonyl oxygen of the *i*th residue and the amide hydrogen of the (*i* + 4)th residue, as revealed in Fig. 2e and g. The depolarized electronic structures around the carbonyl oxygen of the *i*th residue and the amide hydrogen of the (*i* + 4)th residue generally resulted in weaker H-bond energies for the α-helix, as in the WH_alpha_ and ST_alpha_ models, than for the separated H-bonds, as in the MH_alpha_ model. Such depolarizations redistributing the electron density were caused by the local electronic interactions in their neighborhood inside the α-helical structure.

Figure 2f and h show $\mathrm{\Delta \Delta }{\rho }_{\mathrm{HB}}^{{\mathrm{WH}}_{3\_10}-{\mathrm{MH}}_{3\_10}}$ and $\mathrm{\Delta \Delta }{\rho }_{\mathrm{HB}}^{{\mathrm{ST}}_{3\_10}-{\mathrm{MH}}_{3\_10}}$ values for the 3_10_-helical turn 4–2. The $\mathrm{\Delta \Delta }{\rho }_{\mathrm{HB}}^{{\mathrm{WH}}_{3\_10}-{\mathrm{MH}}_{3\_10}}\mathrm{ and \Delta \Delta }{\rho }_{\mathrm{HB}}^{{\mathrm{ST}}_{3\_10}-{\mathrm{MH}}_{3\_10}}$ values indicated depolarization of the oxygen atom of the C=O group at the *i*th residue and the hydrogen atom of the N–H group at the (*i* + 3)th residue in the WH_3_10_ and ST_3_10_ models. However, in contrast to the $\mathrm{\Delta \Delta }{\rho }_{\mathrm{HB}}^{{\mathrm{WH}}_{\mathrm{alpha}}-{\mathrm{MH}}_{\mathrm{alpha}}}$ and $\mathrm{\Delta \Delta }{\rho }_{\mathrm{HB}}^{{\mathrm{ST}}_{\mathrm{alpha}}-{\mathrm{MH}}_{\mathrm{alpha}}}$ values, the $\mathrm{\Delta \Delta }{\rho }_{\mathrm{HB}}^{{\mathrm{WH}}_{3\_10}-{\mathrm{MH}}_{3\_10}}$ values for the 3_10_-helical turn 4–2 were remarkably different from the $\mathrm{\Delta \Delta }{\rho }_{\mathrm{HB}}^{{\mathrm{ST}}_{3\_10}-{\mathrm{MH}}_{3\_10}}$ values, implying other factors besides the helical backbone atoms linking the H-bond pairs that caused the depolarization of the H-bond donor and acceptor in the 4–2 pair. We now discuss why the H-bonds in the ST_3_10_-*n* model were destabilized in comparison to those in the MH_3_10_-*n* model. We found that the C=O group at the *i*th residue and the N–H group at the (*i* + 3)th residue of the H-bond were depolarized in the ST_3_10_-*n* model, as shown in Fig. 2h. This depolarization could be caused by the helical backbone atoms linking the H-bond pair. Whereas the C=O group at the (*i* + 1)th residue was involved in depolarization in α-helices, the C=O group of the H-bond pair was closer to the adjacent N–H group at the (*i* + 2)th residue (2.799 ± 0.033 Å), than to the adjacent C=O group at the (*i* + 1)th residue (3.442 ± 0.025 Å) in the 3_10_-helices. Therefore, in the 3_10_-helices, the adjacent N–H group may cause the depolarization of the H-bond acceptor, resulting in only little destabilization of the H-bond.

## Toward improvement of the H-bond energy by the classical force field

Our calculations for α-helices and 3_10_-helices with the NFA revealed that their H-bond energies are affected by depolarization and polarization due to the local dipole of the neighboring backbone peptide groups. Based on our results, we constructed a model for the classical force field, in which the atomic partial charges (${q}_{\mathrm{N}}^{i}$, ${q}_{\mathrm{H}}^{i}$, ${q}_{\mathrm{C}}^{i}$, and ${q}_{\mathrm{O}}^{i}$) of the N–H and C=O groups of the *i*th peptide group were not constant but were changed by the neighboring peptide groups, respectively.

6 $${q}_{\mathrm{N}}^{i}={q}_{\mathrm{N}}^{0}\left(1-{\delta }_{\mathrm{N}}^{i}\right)$$

7 $${q}_{\mathrm{H}}^{i}={q}_{\mathrm{H}}^{0}\left(1-{\delta }_{\mathrm{H}}^{i}\right)$$

8 $${q}_{\mathrm{C}}^{i}={q}_{\mathrm{C}}^{0}\left(1-{\delta }_{\mathrm{C}}^{i}\right)$$

9 $${q}_{\mathrm{O}}^{i}={q}_{\mathrm{O}}^{0}\left(1-{\delta }_{\mathrm{O}}^{i}\right)$$

where ${q}_{\mathrm{N}}^{0}$, ${q}_{\mathrm{H}}^{0}$, ${q}_{\mathrm{C}}^{0}$, and ${q}_{\mathrm{O}}^{0}$ are the original atomic partial charge, and the amounts of changes in the atomic partial charges of the *i*th peptide group; ${\delta }_{\mathrm{N}}^{i}$, ${\delta }_{\mathrm{H}}^{i}$, ${\delta }_{\mathrm{C}}^{i}$, and ${\delta }_{\mathrm{O}}^{i}$, are functions depending on the backbone structure of the neighboring peptide group. This change may be represented by the interaction energy of the classical mechanics, $U\left({\overrightarrow{\mu }}_{\mathrm{X}}^{i}, {\overrightarrow{\mu }}_{\mathrm{Y}}^{j}\right)$ between the *i*th backbone dipole, ${\overrightarrow{\mu }}_{\mathrm{X}}^{i}$ (X = NH or CO), and the neighboring backbone dipole, ${\overrightarrow{\mu }}_{\mathrm{Y}}^{j}$ (Y = NH or CO) ($j\ne i$). To avoid double counting interaction energies already taken in contributions in the restrained electrostatic potential (RESP) approach (Bayly et al. 1993; Cieplak et al. 1995), the following formula was used to obtain the change in the original atomic partial charge.

10 $${\delta }_{\mathrm{H}}^{i}={\lambda }_{\mathrm{HO}}\left[U\left({\overrightarrow{\mu }}_{\mathrm{NH}}^{i}, {\overrightarrow{\mu }}_{\mathrm{CO}}^{i-1}\right)\right]+{\lambda }_{\mathrm{HH}}\left[U\left({\overrightarrow{\mu }}_{\mathrm{NH}}^{i}, {\overrightarrow{\mu }}_{\mathrm{NH}}^{i-1}\right)\right]$$

11 $${\delta }_{\mathrm{O}}^{i}={\lambda }_{\mathrm{OO}}\left[U\left({\overrightarrow{\mu }}_{\mathrm{CO}}^{i}, {\overrightarrow{\mu }}_{\mathrm{CO}}^{i+1}\right)\right]+{\lambda }_{\mathrm{OH}}\left[U\left({\overrightarrow{\mu }}_{\mathrm{CO}}^{i}, {\overrightarrow{\mu }}_{\mathrm{NH}}^{i+1}\right)\right]$$

where ${\lambda }_{\mathrm{HO}}$, ${\lambda }_{\mathrm{HH}}$, ${\lambda }_{\mathrm{OO}}$, and ${\lambda }_{\mathrm{OH}}$ are fitting parameters. To ensure that the overall charge does not change, local neutrality conditions were imposed, and ${\delta }_{\mathrm{N}}^{i}$ and ${\delta }_{\mathrm{C}}^{i}$ are defined as follows.

12 $${\delta }_{\mathrm{N}}^{i}{q}_{\mathrm{N}}^{0}=-{\delta }_{\mathrm{H}}^{i}{q}_{\mathrm{H}}^{0}$$

13 $${\delta }_{\mathrm{C}}^{i}{q}_{\mathrm{C}}^{0}=-{\delta }_{\mathrm{O}}^{i}{q}_{\mathrm{O}}^{0}$$

**Fig. 3:**
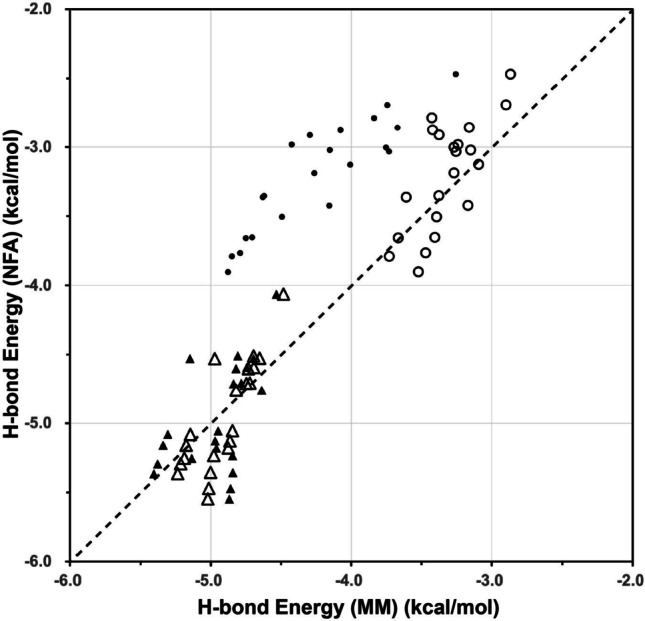
Correlations of the ${E}_{\mathrm{HB}}^{{\mathrm{MM}}_{\mathrm{modified}}}$ and ${E}_{\mathrm{HB}}^{\mathrm{WH}}$ (open symbols), together with those of the ${E}_{\mathrm{HB}}^{\mathrm{MM}}$ and ${E}_{\mathrm{HB}}^{\mathrm{WH}}$ (small filled symbols) for both in the α-helices (circles) and the 3_10_-helices (triangles). The dashed line shows a guide where the longitudinal axis values from NFA have the identical H-bond energies by the MM and MM_modified_ methods. The detail of the latter modification is as follows: the modified H-bond energy ${E}_{\mathrm{HB}}^{{\mathrm{MM}}_{\mathrm{modified}}}=\sum_{ij}\frac{q_i{q}_j}{r_{ij}}+\sum_{ij}\left(\frac{A_{ij}}{r_{ij}^{12}}-\frac{B_{ij}}{r_{ij}^6}\right)$ was computed from the modified charges, *q*_N_, *q*_H_, *q*_C_, and *q*_O_, given by Eqs. (6)–(9) as the changes from the original charges, ${q}_{\mathrm{N}}^0$, ${q}_{\mathrm{H}}^0$, ${q}_{\mathrm{C}}^0$, and ${q}_{\mathrm{O}}^0$, which were −0.4157, 0.2719, 0.5973, and −0.5679, respectively, taken from the AMBER ff99SB force field (Wang et al. 2000). The parameters modifying charges, ${\delta}_{\mathrm{N}}^i$, ${\delta}_{\mathrm{H}}^i$, ${\delta}_{\mathrm{C}}^i$, and ${\delta}_{\mathrm{O}}^i$, in Eqs. (6)–(9) were computed by Eqs. (10)–(13), where the fitting parameters *λ*_HO_, *λ*_HH_, *λ*_OO_, and *λ*_OH_ were the coefficients of the interaction energies between the *i*th backbone dipole, ${\overrightarrow{\mu}}_{\mathrm{X}}^i$ (X = HN or CO), and the neighboring backbone dipole, ${\overrightarrow{\mu}}_{\mathrm{Y}}^j$ (Y = HN or CO) (*j* ≠ *i*), as in Eqs. (10) and (11): $U\left({\overrightarrow{\mu}}_X^i,{\overrightarrow{\mu}}_Y^j\right)=\frac{\overrightarrow{\mu_X^i}\overrightarrow{\mu_Y^j}}{r_{ij}^3}-\frac{3\left({\overrightarrow{\mu}}_X^i{\overrightarrow{r}}_{ij}\right)\left({\overrightarrow{\mu}}_Y^j{\overrightarrow{r}}_{ij}\right)}{r_{ij}^5}$. Here, *r*_*ij*_ is the distance between the center of the *i*th backbone dipole and that of the *j*th dipole. The local dipole moments were given as ${\overrightarrow{\mu}}_{\mathrm{C}\mathrm{O}}^k=\frac{1}{2}\left({q}_{\mathrm{C}}^k-{q}_{\mathrm{O}}^k\right)\left({\overrightarrow{r}}_{\mathrm{C}}^k-{\overrightarrow{r}}_{\mathrm{O}}^k\right)$ and ${\overrightarrow{\mu}}_{\mathrm{H}\mathrm{N}}^k=\frac{1}{2}\left({q}_{\mathrm{H}}^k-{q}_{\mathrm{N}}^k\right)\left({\overrightarrow{r}}_{\mathrm{H}}^k-{\overrightarrow{r}}_{\mathrm{N}}^k\right)$ (*k* = *i* or *j*), as indicated in Kondo et al. (2019). The four parameters *λ*_HO_, *λ*_HH_, *λ*_OO_, and *λ*_OH_ were determined by minimizing the square of the difference between ${E}_{\mathrm{HB}}^{{\mathrm{MM}}_{\mathrm{modified}}}$ and ${E}_{\mathrm{HB}}^{\mathrm{WH}}$ simultaneously for H-bond energies of 21 WH_alpha_ values of α-helices (Kondo et al. 2019) and for those of 21 WH_3_10_ values of 3_10_-helices (Kondo et al. 2022). The resulted parameters were *λ*_HO_ = −11.535, *λ*_HH_ = 38.517, *λ*_OO_ = 16.967, and *λ*_OO_ = 4.587, respectively, where the unit was all (kcal/mol)

We determined the parameters ${\lambda }_{\mathrm{HO}}$, ${\lambda }_{\mathrm{HH}}$, ${\lambda }_{\mathrm{OO}}$, and ${\lambda }_{\mathrm{OH}}$, by using the H-bond energies calculated with NFA, ${E}_{\mathrm{HB}}^{\mathrm{WH}}$, for both α-helices and 3_10_-helices, and those of the AMBER ff99SB force field parameters (Wang et al. 2000), ${E}_{\mathrm{HB}}^{\mathrm{MM}}$. Using the determined fitting parameters, we modified the MM values of the H-bond energies, denoted as ${E}_{\mathrm{HB}}^{{\mathrm{MM}}_{\mathrm{modified}}}$, and plotted the ${E}_{\mathrm{HB}}^{{\mathrm{MM}}_{\mathrm{modified}}}$ versus ${E}_{\mathrm{HB}}^{\mathrm{WH}}$ (open symbols), together with the original ${E}_{\mathrm{HB}}^{\mathrm{MM}}$ versus ${E}_{\mathrm{HB}}^{\mathrm{WH}}$ (small filled symbols) in Fig. 3. The root mean square deviation of the original ${E}_{\mathrm{HB}}^{\mathrm{MM}}$ from the ${E}_{\mathrm{HB}}^{\mathrm{WH}}$ values was evaluated to be 0.78 kcal/mol, and that of the ${E}_{\mathrm{HB}}^{{\mathrm{MM}}_{\mathrm{modified}}}$ from the ${E}_{\mathrm{HB}}^{\mathrm{WH}}$ values was calculated to be 0.28 kcal/mol, much smaller than the original one. It suggests that this modification of the classical MM force field could well approximate the H-bond energies provided by NFA. In particular, those for the α-helices were remarkably improved, as shown in Fig. 3. For extended structures, the amounts of changes in the atomic partial charges should be small because the dipole–dipole interaction quickly decreases in inverse proportion of the cube of the distance between the dipole pair.

## Concluding remarks

We have briefly reviewed our recent computational studies of hydrogen bonds (H-bonds) in helical secondary structures of proteins, α-helix and 3_10_-helix, using a Negative Fragmentation Approach (NFA) with density functional theory (DFT). Our computation showed that the H-bond energies of the α-helix are generally weaker than those of the separated H-bonds due to the depolarized electronic structures around the carbonyl oxygen of the *i*th residue and the amide hydrogen of the (*i* + 4)th residue. The H-bond energies of the 3_10_-helix are also weaker than those of the separated H-bonds, but the effects are not so large as those for the α-helix. Whereas the adjacent C=O group is involved in the depolarization of the H-bond acceptor in α-helices, the C=O group of the H-bond pair is closer to the adjacent N–H group than to the adjacent C=O group in the 3_10_-helices. Therefore, the weak destabilization of the H-bond is attributed to the balance of the depolarization and polarization caused by the adjacent N–H group and the C=O group. Based on the findings from our computational results, a model was constructed in which the atomic partial charges of the N–H and C=O groups of the backbone peptide groups forming H-bonds are changed by the neighboring peptide groups, respectively. This modified MM model well reproduced the H-bond energies of α-helices and 3_10_-helices given by the NFA computation. We expect that this modification could lead to more reliable MD simulations in the future.

## Electronic supplementary material

Below is the link to the electronic supplementary material.

(DOCX 2.65 MB)

## Data Availability

The data that support the findings of this study are available from the corresponding author upon reasonable request.
