# Supplementary material for: Quantum chemical studies on hydrogen bonds in helical secondary structures
Source: Biophys Rev. 2023 Jan 6;14(6):1369–78. doi: 10.1007/s12551-022-01034-5 (PMC9842822; doi:10.1007/s12551-022-01034-5)
Supplement: Supplementary file 1 — (DOCX 2.65 MB) [file 12551_2022_1034_MOESM1_ESM.docx]

Supporting information

**“Quantum chemical studies on hydrogen bonds in helical secondary structures”**

Yu Takano,^1,2^* Hiroko X. Kondo,^1,3,4^ Haruki Nakamura^2^

1 Graduate School of Information Sciences, Hiroshima City University, Hiroshima 731-3194, Japan

2 Institute for Protein Research, Osaka University, Suita 565-0871, Japan

3 Faculty of Engineering, Kitami Institute of Technology, Kitami 090-8507, Japan

4 RIKEN Center for Biosystems Dynamics Research, Suita 565-0874, Japan

**
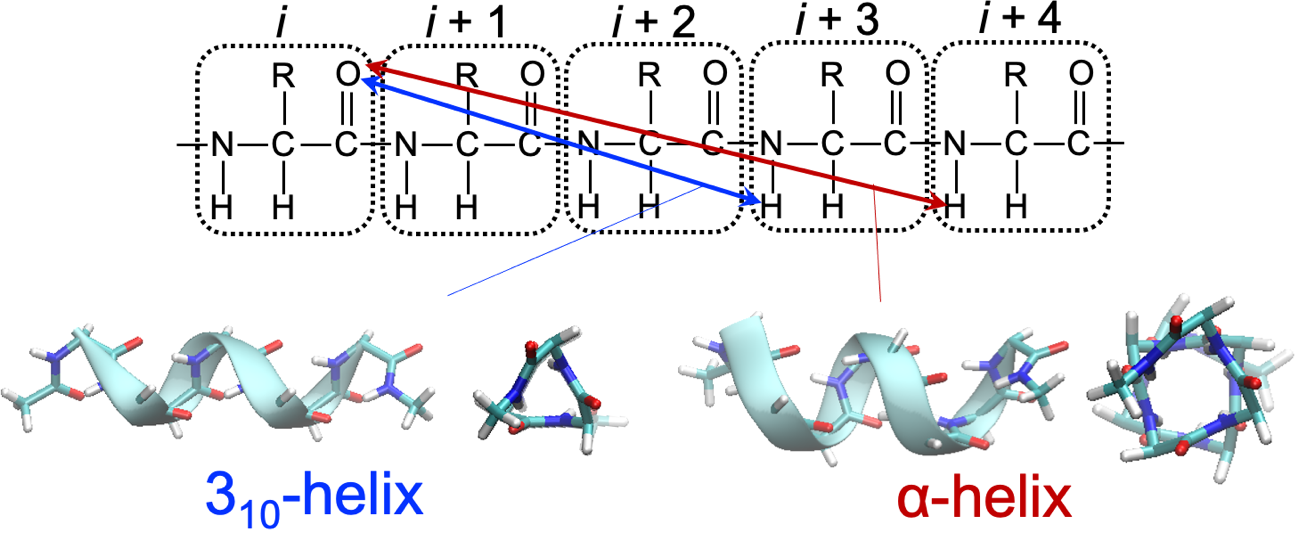
**

**Fig. S1**. Schematic hydrogen bonding patterns and molecular structures of 3_10_-helix and α-helix.

**Table S1.** The intercept, the slope, and the correlation coefficient of the linear regressions drawn in Figs. 2a and 2b.

| Helix | Model | Intercept | Slope | Correlation coefficient |
| --- | --- | --- | --- | --- |
| α-helix | WH | 0.303 | 0.792 | 0.88 |
|  | ST | 0.185 | 0.787 | 0.99 |
|  | MM | 0.233 | 1.01 | 0.98 |
| 3_10_-helix | WH | 7.014 | 2.15 | 0.85 |
|  | ST | -1.672 | 0.616 | 0.83 |
|  | MM | 2.812 | 1.393 | 0.88 |
